# Assessing Statistically Significant Heavy-Metal Concentrations in Abandoned Mine Areas via Hot Spot Analysis of Portable XRF Data

**DOI:** 10.3390/ijerph14060654

**Published:** 2017-06-18

**Authors:** Sung-Min Kim, Yosoon Choi

**Affiliations:** 1 Energy Resources Institute, Pukyong National University, Busan 48513, Korea; snuhyrule@hanmail.net; 2 Department of Energy Resources Engineering, Pukyong National University, Busan 48513, Korea

**Keywords:** hot spot analysis, Getis-Ord Gi*, portable X-ray fluorescence, soil contamination, GIS

## Abstract

To develop appropriate measures to prevent soil contamination in abandoned mining areas, an understanding of the spatial variation of the potentially toxic trace elements (PTEs) in the soil is necessary. For the purpose of effective soil sampling, this study uses hot spot analysis, which calculates a *z*-score based on the Getis-Ord Gi* statistic to identify a statistically significant hot spot sample. To constitute a statistically significant hot spot, a feature with a high value should also be surrounded by other features with high values. Using relatively cost- and time-effective portable X-ray fluorescence (PXRF) analysis, sufficient input data are acquired from the Busan abandoned mine and used for hot spot analysis. To calibrate the PXRF data, which have a relatively low accuracy, the PXRF analysis data are transformed using the inductively coupled plasma atomic emission spectrometry (ICP-AES) data. The transformed PXRF data of the Busan abandoned mine are classified into four groups according to their normalized content and *z*-scores: high content with a high *z*-score (HH), high content with a low *z*-score (HL), low content with a high *z*-score (LH), and low content with a low *z*-score (LL). The HL and LH cases may be due to measurement errors. Additional or complementary surveys are required for the areas surrounding these suspect samples or for significant hot spot areas. The soil sampling is conducted according to a four-phase procedure in which the hot spot analysis and proposed group classification method are employed to support the development of a sampling plan for the following phase. Overall, 30, 50, 80, and 100 samples are investigated and analyzed in phases 1–4, respectively. The method implemented in this case study may be utilized in the field for the assessment of statistically significant soil contamination and the identification of areas for which an additional survey is required.

## 1. Introduction

Mining is a global industry that can be hazardous to public health and safety, and that can cause damage to the surrounding environment, including the land, soil, water, and forest [1]. Soil contamination is a significant problem among the various mining hazards, because mine waste generally contains a higher content of potentially toxic trace elements (PTEs) than regular industrial waste [2]. Elevated PTE levels may be found in and around mines because of the dispersion of mine waste down-slope due to surface runoff, wind action, and effluent drainage into nearby soil and open water systems [3]. An anomalous PTE concentration may affect the soil environment and quality, ultimately posing a serious risk to crops, livestock, and human health through food chain accumulation [4]. Therefore, the spatial variation of PTEs in both operating and abandoned mining areas should be investigated, so as to identify the appropriate isolation or treatment measures to prevent soil contamination [5,6].

Many studies on PTE spatial variation have been conducted worldwide, in which this variation has been explored and mapped using geostatistical interpolation methods in geographic information systems (GIS) [7,8,9,10,11,12]. These methods are valuable tools for understanding PTE spatial variation and generating soil contamination maps. In these techniques, the spatial autocorrelation of the studied phenomenon is considered in order to create spatial correlation structures and to estimate the unknown values at unsampled locations [13]. With the development of a portable X-ray fluorescence (PXRF) instrument, which is relatively more cost and time effective than inductively coupled plasma atomic emission spectrometry (ICP-AES), the type and content of PTEs in soil can be investigated at a higher number of sampling points. The utilization of larger datasets can be a significant advantage for spatial variation estimations. PXRF analysis data have been used as input data to explore PTE spatial variation using geostatistical methods in previous studies [14,15,16]. However, the generation of high-quality soil contamination maps remains difficult because of the relatively low accuracy of PXRF analysis data. Therefore, Lee et al. [2] have attempted to use both ICP-AES and PXRF analysis data for geostatistical interpolation, so as to compensate for any disadvantages of these instruments while incorporating their advantageous features. Although such geostatistical interpolation results are useful for predicting PTE concentrations in mining areas for which sampling was not conducted, these methods cannot provide information on whether the samples are statistically significant. For instance, kriging, which is a widely used geostatistical interpolation method, uses the data covariance to predict unknown values and quantifies the uncertainty in the estimation of those values [17]. However, the kriging variance is zero at the observations and the predicted values of the observations are exactly the same as the observed values. Thus, this method cannot aid in the assessment of the sampling reliability because it regards the observations as accurate.

To overcome this limitation, hot spot analysis, which identifies a statistically significant hot spot sample by calculating *z*-scores based on the Getis-Ord Gi* statistic [18], is used in this study. In this system, a feature with a high value is interesting; however, it may not correspond to a statistically significant hot spot. For a feature to be recognized as a statistically significant hot spot, it must have a high value and must also be surrounded by other high-value features. The Getis-Ord Gi* statistic is calculated by comparing the sum of a point and its nearest neighbors to the sum of all points in a given study area. This approach has been widely used in applied geographic research to identify the clustering of species populations [19], disease [20], crime incidence [21], medical care availability [22], and food retailers [23]. However, few studies applying hot spot analysis to PTE distributions have been conducted.

Among those studies, McClintock [24] recently utilized hot spot analysis to evaluate the risk of lead contamination in post-industrial landscapes in Oakland, California. Getis-Ord Gi* analysis revealed that the lead contamination in that region is related to the land-use history on the city and neighborhood scales. However, although the McClintock study [24] identified contaminated hot spot areas by analyzing the statistical significance of various soil samples, the results were only utilized to obtain the result of the soil survey, and could not provide information to support the ongoing soil survey process. In addition, Griffith et al. [25] have considered the impact of location errors on local spatial autocorrelation by simulating significant heavy metal clusters using local Moran’s I and Getis-Ord Gi* statistics. That study confirmed that more severe deviations from the true results are observed if the samples contain larger location errors. This result elucidates the importance of spatial location accuracy in hot spot analysis. However, as previously mentioned, soil survey planning could not be supported by the hot spot analysis results in the Griffith et al. study [25].

The aim of the present study is to assess statistically significant soil contamination in a given region and to support soil survey planning by determining areas for which an additional survey is required during the sampling process. Getis-Ord Gi* hot spot analysis is employed and a group classification method is proposed to identify suspect samples requiring a complementary survey. Element analysis data from PXRF instruments located at the abandoned Busan mine in Korea are used for analysis. As PXRF instruments can investigate a considerably greater number of sampling points more rapidly than the alternatives, they are useful for rapid planning in a soil survey process. To calibrate the PXRF data, which tend to overestimate the PTE concentrations in the study area, the PXRF analysis data are transformed using the ICP-AES data. However, a geochemical investigation includes inevitable errors in the data analysis, which stem from a variety of sources. Therefore, it is important to identify problems in the geochemical data to obtain higher-accuracy results, which in turn facilitate a better understanding of the soil contamination in the examined area. The results indicate that information on the statistical significance of each sample can be obtained via hot spot analysis. Hence, critical areas in soil sampling can be identified and the next soil sampling process can be planned.

## 2. Materials and Methods

### 2.1. Study Area and Soil Sampling

**Figure 1:**
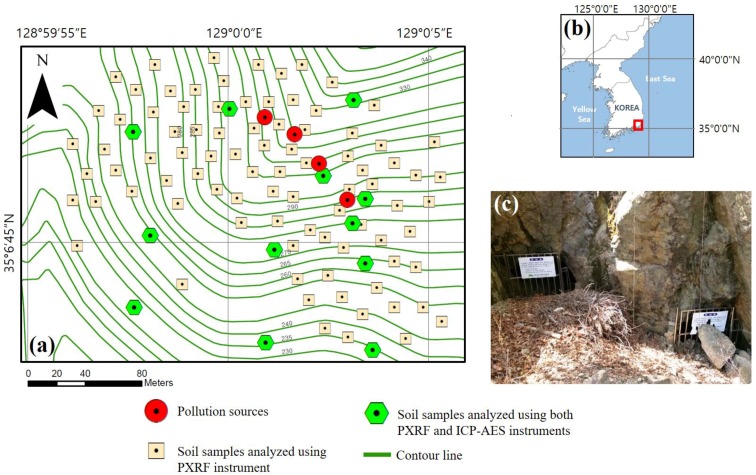
Study area: (**a**) Locations of pollution sources and soil sampling points for ICP-AES and PXRF analyses; (**b**) Study area location in Korea; (**c**) Photograph of closed pit heads at the Busan mine.

The currently abandoned Busan mine located at Saha-gu, Busan, South Korea, was selected as the target area (Figure 1). The Busan mine was in operation until 1986 and produced a total of 2246 tons of iron [26]. Although high PTE concentrations have been found around the waste rock pile and pit heads, no formal environmental treatment of their surroundings has been implemented [26]. In addition, the dispersion of mine waste rocks and tailings via surface erosion has caused soil contamination [2]. The extent of the target area was defined as 280 m × 200 m considering the location and dispersion of pollution sources based on the topography. Soil sampling was planned for a total of 100 points according to a four-phase procedure. To support the sampling-point planning for each phase, hot spot analysis and the proposed group classification method were employed. The sampling-point density was relevant for the purpose of this study, and a sample was taken in each 560 m^2^ area.

A dataset obtained via on-site analysis of the copper content was used for hot spot analysis in this study. The dataset was acquired using a PXRF instrument (Innov-X DELTA Handheld XRF analyzer; Olympus, Japan) equipped with a gold anode as the excitation source and a silicon drift detector. This PXRF instrument operates at 40 kV and 0.1 mA. Using a hand auger, surface soil samples were taken down to 10 cm in depth at various sampling points. The samples were comprised of a composite of nine subsamples taken within a 5 m × 5 m area. After the soil samples were disaggregated and sieved to <2 mm as loose powders in the field, they were analyzed using PXRF. The element analysis results yielded by the PXRF instrument can vary depending on the water content of the soil [27,28]. Tolner et al. [28] determined that approximate reductions of 1–3%, 23–30%, and 30–39% in the detected metallic elements are obtained when the water content is 10%, 15%, and 20%, respectively, compared to the results obtained for completely dry soil. Therefore, in this study, the PXRF element analysis was conducted when the soil-sample water content was less than 10% [8,9]. The water content was measured using a portable soil moisture meter (PMS-714; Lutron, Taiwan). The PXRF instrument employs the fundamental parameters (FP) method to support quantitative analysis. The FP method has associated stored libraries and allows elemental analysis to be performed without standards or calibration curves. The accuracy of the results can be improved if well-prepared samples are available for calibration.

For the purpose of calibration, the correlation between the PXRF and ICP-AES (VISTA-PRO; Varian, Palo Alto, CA, USA) analysis data was examined based on the data obtained at 12 sampling points, for which both PXRF and ICP-AES analyses were performed. The ratio of the validation data to the sampling data was 12% in this study, considering the expense budget. This ratio is similar to that (12.5%) of a previous study [16] in which the PXRF (training set) and ICP-AES data (validation set) were compared. For the ICP-AES analysis, after air-drying at 25 °C in the laboratory for 5 d, the soil samples were disaggregated and sieved to particles of less than 2 mm in size, before being ground to a fine powder (<2 μm). According to the Korean standard test (KST) method for the chemical analysis of soils [29,30], the soil samples were digested with 0.1 N of HCl solution, with 10 g of soil in 50 mL of the solution. At an approximate ratio of 3:1, concentrated HCl (21 mL) and HNO_3_ (7 mL) (aqua regia) were added to 2-g soil samples. The samples were then heated at 60 °C for 30 min and subsequently at 140 °C for 90 min. After cooling and filtration, the solutions were diluted to 100 mL with distilled water. The copper was quantified via ICP-AES using the measured intensity of the atomic emission at a wavelength of 324.75 nm and a calibration curve according to the KST method. A blank test was conducted for the calibration.

### 2.2. Hot Spot Analysis

Spatial correlation and autocorrelation are very important in spatial modeling, and various methods for testing and measuring spatial autocorrelation have been developed. Moran’s *I* is one of the best-known spatial autocorrelation measures used by geographers, while the semi-variance is the most popular tool used by geologists and remote sensing analysts [31]. For spatial econometricians, the typical approach involves an estimation of the spatial autocorrelation coefficients of regression equations [32], which are applied globally to the entire study area. However, it is often necessary to examine a pattern at a local scale, particularly if the process is spatially nonstationary. Thus, Getis and Ord [18] focused upon local effects to develop a spatial association measure called the “Getis-Ord Gi* statistic.”

In this study, the Getis-Ord Gi* statistic was calculated for each feature in a given dataset using the Hot Spot Analysis (Getis-Ord Gi*) tool in GIS software ArcMap 10.1 (Environmental Systems Research Institute, Inc., Redlands, CA, USA). To constitute a statistically significant hot spot, a feature with a high value should also be surrounded by other features with high values, as noted above. The Getis-Ord Gi* statistic is calculated by comparing the local sum of the value for the feature in question and those of its neighbors to the sum of all feature values, such that:

(1) $$G_{i}^{*}=\frac{\sum_{j=1}^{n}w_{i,j}x_{j}-\bar{X}\sum_{j=1}^{n}w_{i,j}}{S\sqrt{\frac{\left[n\sum_{j=1}^{n}w_{i,j}^{2}-{\left(\sum_{j=1}^{n}w_{i,j}\right)}^{2}\right]}{n-1}}}$$

where $x_{j}$ is the attribute value for feature j, $w_{i,j}$ is the spatial weight between features i and j, n is the total feature number, and:

(2) $$\bar{X}=\frac{\sum_{j=1}^{n}x_{j}}{n}$$

(3) $$S=\sqrt{\frac{\sum_{j=1}^{n}x_{j}^{2}}{n}}-{\left(\bar{X}\right)}^{2}$$

The resultant Gi* statistic is a *z*-score. For statistically significant positive *z*-scores, the larger the *z*-score, the more intense the clustering of high values (i.e., a hot spot is obtained). For statistically significant negative *z*-scores, the smaller the *z*-score, the more intense the clustering of low values (i.e., a cold spot is obtained). When the local sum is very different from the expected local sum and that difference is too large to be the result of random chance, the *z*-score is statistically significant.

### 2.3. Methods of Applying Hot Spot Analysis to Copper-Concentration Sampling and Assessment

To assess statistically significant soil contamination and to determine the areas in which an additional survey is required, the PXRF data for the Busan abandoned mine study area were classified into four groups according to the concentrations and the *z*-scores yielded by the Gi* statistics. The four groups were as follows: a high concentration value with a high *z*-score (HH; class 1); a low concentration value with a high *z*-score (LH; class 2); a low concentration value with a low z-score (LL; class 3); and a high concentration value with a low z-score (HL; class 4). To classify the dataset elements into these four groups, a scatter plot was created in which the concentration value normalized by the standard deviation (std. dev.) and the Gi* *z*-score were utilized as the *x* and *y* variables, respectively. In statistics, the normalized copper concentration value and Gi* *z*-score correspond to the signed number of standard deviations with a dimensionless quantity. The dataset elements are classified into four groups based on the value of 0 as a criterion for both the *x* and *y* variables. Note that the classification of samples as HL and LH may result from measurement errors, and these regions require a complementary survey.

The soil sampling was conducted according to a four-phase procedure in which the hot spot analysis and proposed group classification method were employed to support the development of a sampling plan for the following phase. For each phase, soil samples were obtained and analyzed by the PXRF instrument. The entire study area was considered and covered in all four phases. Overall, 30, 50, 80, and 100 sampling points were utilized in phases 1–4, respectively. The group classification method was applied to the samples obtained in each phase so as to identify the areas requiring a complementary survey in the next phase. Furthermore, the hot spot analysis results indicated the areas of significant soil contamination. Such hot spot areas require careful consideration in the context of both the soil sampling plan and the reclamation plan. To achieve the aim of applying the proposed method, a total of 100 sampling points were investigated as an initial plan in this study. It seems that the results of these 100 sampling points are relevant for a contamination assessment of the study area. That is, if significant indications of contamination are obtained in the final results, continuous soil sampling can be conducted until no noteworthy results are found.

To confirm the appropriate method of spatial relationship conceptualization for the hot spot analysis, four methods were used to examine the copper concentrations: the inverse distance method, the inverse distance squared method, the fixed distance band method, and the zone of indifference method [33].

## 3. Results and Discussion

### 3.1. Soil Sampling Results

**Figure 2:**
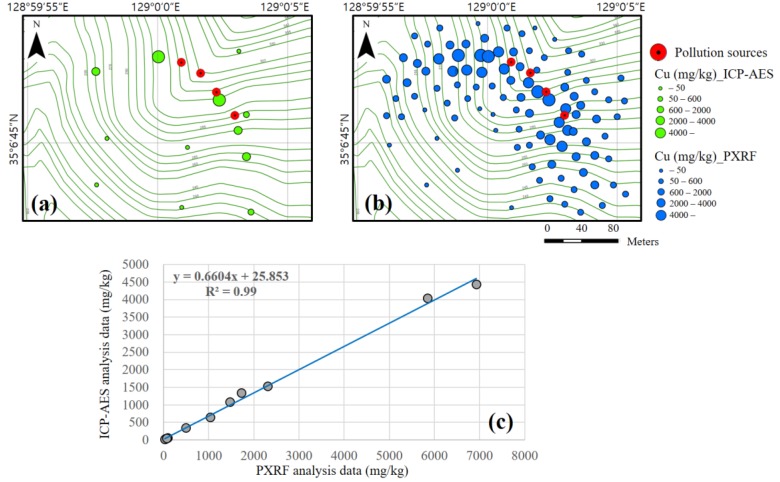
Element analysis results: Copper concentrations as analyzed using (**a**) ICP-AES and (**b**) PXRF; (**c**) Correlation between ICP-AES and PXRF analysis data.

Figure 2a,b shows the spatial distributions of the copper concentration as determined by the ICP-AES and PXRF instruments, respectively. Samples with copper concentrations of more than 50 mg/kg were obtained in most areas, and high concentrations were detected near the pollution sources. It has been reported that different living organisms experience severe toxicity effects in response to copper concentrations of approximately 50 mg/kg, and no biomass is produced at levels of approximately 600 mg/kg [34]. A very strong correlation was obtained between the datasets yielded by the PXRF and ICP-AES, with an *R*^2^ value of 0.99, as shown in Figure 2c. The copper concentrations at the 12 sampling points analyzed using both the ICP-AES and PXRF instruments are listed in Table 1. Because the ICP-AES analysis data have a relatively high accuracy, the PXRF analysis data were transformed by calculating the trend equations of these two datasets. These transformed PXRF results are also listed in Table 1. It should be noted that the transformation overestimates the values with low concentrations, although the PXRF values were properly transformed for concentrations of more than 80 mg/kg. The transformed PXRF analysis data based on the correlation were used to implement the hot spot analysis and to generate copper contamination maps.

### 3.2. Spatial Autocorrelation Results

**Figure 3:**
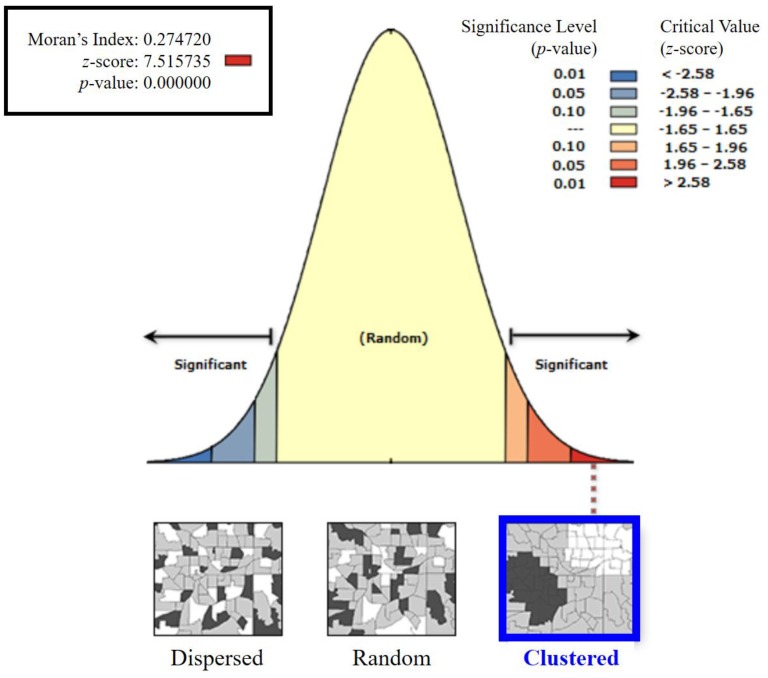
Global Moran’s I statistic results yielded by the Spatial Autocorrelation tool in ArcMap 10.1. The tendency of the feature pattern obtained in the study area toward clustering is apparent.

Prior to the implementation of the hot spot analysis, the spatial autocorrelation results for the net samples based on the feature locations and attribute values were obtained, as shown in Figure 3. These results were derived from the Global Moran’s I statistic of the Spatial Autocorrelation tool provided in ArcMap 10.1. The Spatial Autocorrelation tool evaluates whether the feature pattern is clustered, dispersed, or random. Positive and negative Moran’s I index values indicate a tendency toward clustering and dispersion, respectively. The *z*-scores and *p*-values were calculated as Global Moran’s I statistics to indicate whether or not the null hypothesis could be rejected. In this study, the null hypothesis was complete spatial randomness, in which case the feature values would be randomly distributed across the study area. This null hypothesis must be rejected if statistically significant clustering is to be obtained, which evidences the underlying spatial precondition employed in the hot spot analysis. In this study, the *z*-score was found to be 7.52 and there was a less than 1% likelihood that this clustered pattern could be the result of random chance (Figure 3).

**Figure 4:**
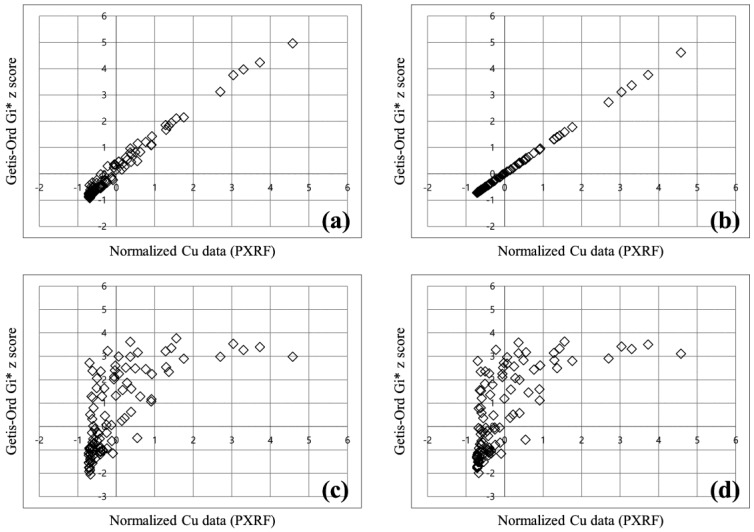
Scatter plots showing the relationship between a normalized copper concentration and Gi* *z*-score for different spatial relationship conceptualization methods: (**a**) Inverse distance method; (**b**) inverse distance squared method; (**c**) fixed distance method; and (**d**) zone of indifference method.

Figure 4 shows scatter plots of the results obtained from the final 100 samples considered in phase 4 of the PXRF analysis (discussed in more detail in Section 3.3) according to the different spatial relationship conceptualization methods. Here, the *x* and *y* variables are the copper concentration value normalized by the std. dev. and the Gi* *z*-score, respectively. The normalized copper concentration of each sample was calculated by dividing the difference between the sample and mean values by the std. dev. This is a widely used method for the standardization of a raw value in statistics. As shown in Figure 4, the Gi* *z*-scores yielded by the inverse distance method and the inverse distance squared method are almost identical to the normalized concentration values (Figure 4a,b). This indicates that the hot spot analysis results based on these methods cannot provide much additional information beyond that yielded by the concentration value. On the other hand, the *z*-scores of the fixed distance band method and the zone of indifference method provide information beyond that given by the concentration value (Figure 4c,d). It is generally accepted that the fixed distance band method is an excellent tool for conceptualizing spatial relationships [33]. In this method, the analysis scale does not change across the study area, because a critical fixed distance is used to select the neighbors included in the analysis. Therefore, the fixed distance band method was utilized in this study to assess the statistically significant soil contamination and to determine areas requiring an additional survey.

### 3.3. PXRF Analysis and Classification

**Figure 5:**
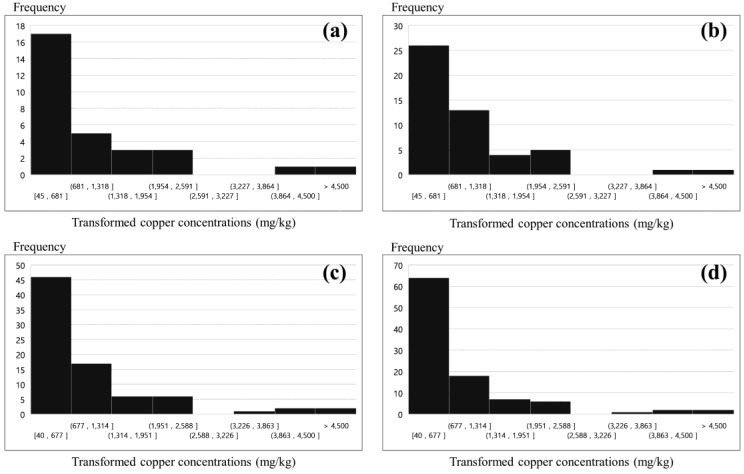
Copper-concentration histograms for transformed PXRF data: (**a**) 30 (first phase); (**b**) 50 (second phase); (**c**) 80 (third phase); and (**d**) 100 sampling points (fourth phase).

**Figure 6:**
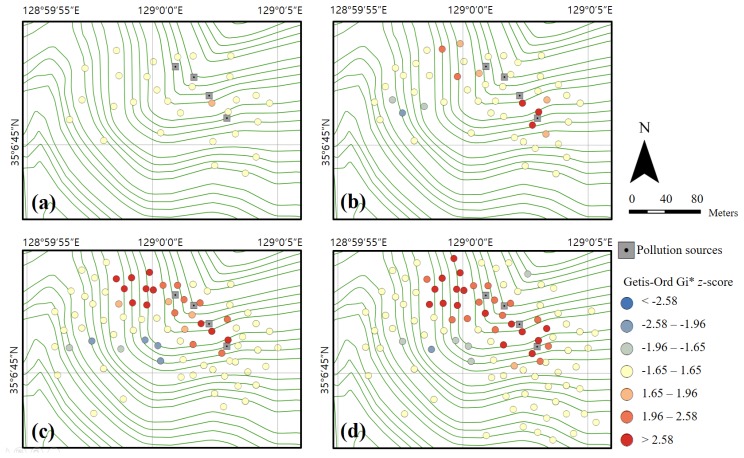
Hot spot analysis maps for copper in each phase: (**a**) 30 (first phase); (**b**) 50 (second phase); (**c**) 80 (third phase); and (**d**) 100 sampling points (fourth phase).

**Figure 7:**
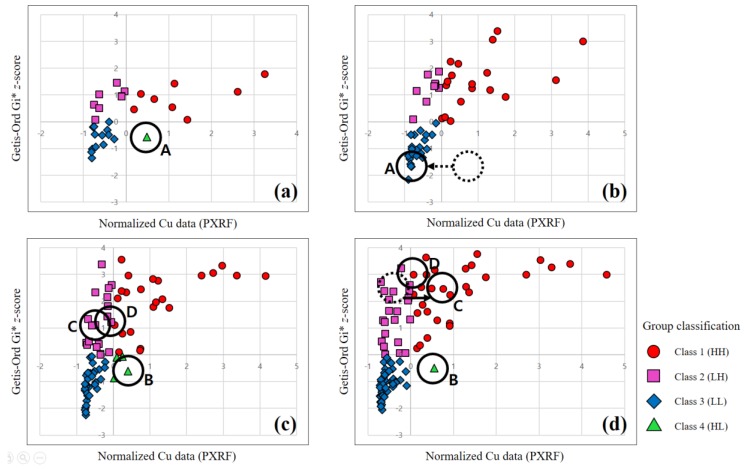
Group classification according to the copper concentration and Gi* *z*-score for each phase: (**a**–**d**) Phases 1–4, respectively. Samples A and B were estimated to be erroneous and a re-investigation was conducted for those sampling points.

**Figure 8:**
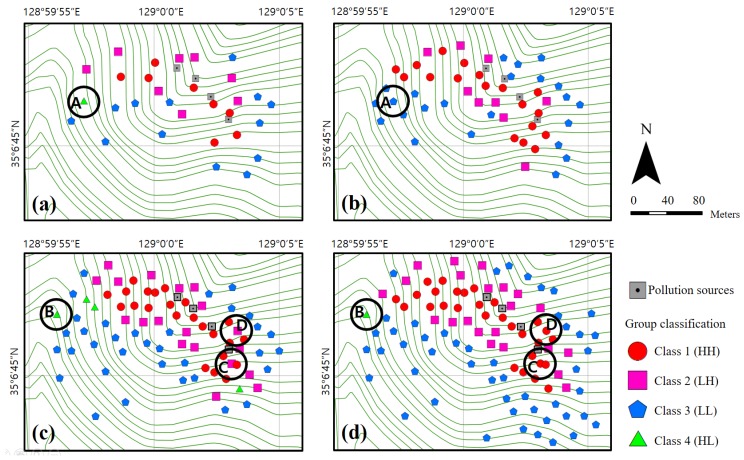
Classified sample distribution maps for each phase: (**a**–**d**) Phases 1–4, respectively. Samples A and B were estimated to be erroneous and a re-investigation was conducted for those sampling points.

As the first phase of the PXRF analysis and classification process, soil sampling was randomly conducted for 30 sampling points located in the study area. The samples were then analyzed using PXRF. The copper concentration histogram obtained for these 30 samples is presented in Figure 5a, in which the distribution is positively skewed. Note that hot spot analysis can be applied to skewed data provided that each feature is associated with several neighbors, and under the assumption that the distribution is asymptotically normal [35]. Figure 6a shows the spatial distribution of the Gi* *z*-scores resulting from the hot spot analysis. In the first phase, although one sample near the pollution sources exhibits a slightly high Gi* *z*-score, the hot spot is not remarkable; this lack of distinction results from a lack of data. The statistical summary of the first phase is as follows. The transformed PXRF analysis data yielded minimum, maximum, mean, and std. dev. values of 45, 4602, 961.6, and 1121.5 mg/kg for the copper concentration, respectively. For the Gi* *z*-scores, minimum, maximum, mean, and std. dev. values of −1.36, 1.78, 0.18, and 0.86 were obtained via hot spot analysis, respectively. The results are listed in Table 2. Figure 7a shows the scatter plot of the normalized copper concentrations and Gi* *z*-scores of the first phase. The normalized copper content was determined by dividing the difference between each sample value and mean value by the std. dev. Hence, the 30 samples of the first phase were classified into four groups (classes 1–4, as defined above). The *x*- and *y*-axes were used to split the criteria. In other words, the samples were classified based on four distinct quadrants. Classes 1 and 3, corresponding to HH and LL, respectively, were regarded as containing explicit hot and cold spots, respectively. Class 2 (LH) was regarded as containing probable hot spots, despite the relatively low content values of these samples. Similarly, Class 4 (HL) was regarded as containing probable cold spots, despite the relatively high content values of these samples. However, the classification of samples into classes 2 and 4 may result from some error in the investigation, as noted above. Figure 8a illustrates the distribution of the classified samples over the study area for phase 1. The class-1 samples are clustered near the pollution sources, whereas the class-2 samples are distributed around the class-1 samples. It is noteworthy that the sample marked A in this figure, which is only classified as class 4, is located far from the pollution source. Because 30 sampling points are insufficient to determine the characteristics of the study area, additional surveys were conducted for the second phase. This phase focused on the areas near classes 1 and 2 and sample A.

As the second phase, soil sampling was conducted on 20 additional sampling points, with the samples again being analyzed using PXRF. In addition, a re-analysis of the sample-A sampling point was conducted, which confirmed that there was an error in the first measurement. The first and second copper concentration measurements for sample A, which was reclassified as class 3, are listed in Table 3. In other words, sample A, for which a measurement error was suspected, was re-analyzed and identified as an explicit cold spot. The copper concentration histogram obtained for the 50 samples examined in phase 2 is shown in Figure 5b. As in the first phase, the distribution is positively skewed. Figure 6b shows the spatial distribution of the Gi* *z*-scores obtained via hot spot analysis. Here, the hot and cold spots are more remarkable compared to the first-phase results. The statistical summary of the second phase results is as follows. The transformed PXRF analysis data yielded minimum, maximum, mean, and std. dev. values of 45, 4602, 902.8, and 958.0 mg/kg for the copper concentration, respectively. The Gi* *z*-scores yielded minimum, maximum, mean, and std. dev. values of −2.16, 3.38, 0.29, and 1.42, respectively, via hot spot analysis (Table 2). Figure 7b shows a scatter plot of the normalized copper concentrations and Gi* *z*-scores of the second phase. As seen previously, the 50 samples of the second phase were classified into four groups. The classification is more obvious than in the first phase, because the Gi* *z*-scores are more widely spread as a result of the larger dataset. Figure 8b shows the distribution of the classified samples in the study area. Here, class-1 samples are clustered near the pollution sources and spread to the west and south of these sources. Additional surveys for the third phase were mostly conducted in the western and central parts of the study area, for convenience and considering the topography.

As the third phase, 30 additional sampling points were analyzed using PXRF. The copper concentration histogram for the 80 samples is shown in Figure 5c, and a positive skew is again apparent for this distribution, as in the previous phases. Figure 6c shows the spatial distribution of the Gi* *z*-scores resulting from the hot spot analysis. The hot spots around the pollution sources are clearly distinguishable. In particular, the area west of the pollution sources holds a remarkable hot spot zone. The transformed PXRF analysis data yielded minimum, maximum, mean, and std. dev. values of 40, 5477, 895.6, and 1102.2 mg/kg for the copper concentration, respectively. The Gi* *z*-scores yielded minimum, maximum, mean, and std. dev. values of −2.26, 3.56, 0.41, and 1.61, respectively, as obtained via hot spot analysis (Table 2). Figure 7c shows a scatter plot of the 80 samples classified into the four groups. It is notable that four samples are classified as class 4. However, three of these four samples (excluding the sample labeled B in the figure) are located near the *x*- or *y*-axes. Thus, although these samples are classified as class 4, they may not be important because they can be classified to another class by slight variations of variables. As shown in Figure 8c, these class-4 samples are located on the boundary between the class-1 (explicit hot spot) and class-3 (explicit cold spot) regions. Further, samples C and D, which are classified as class 2, are surrounded by class-1 samples (Figure 8c). In addition, samples C and D have relatively high Gi* *z*-scores compared to the concentrations (Figure 7c). Therefore, for the fourth phase, a re-analysis of samples B, C, and D was planned.

In the fourth phase, 20 additional sampling points were analyzed using PXRF. Most of these points were located in the southeastern part of the study area. The sampling points of samples B, C, and D were re-investigated and the results are listed in Table 3. As a result of this re-analysis, samples B and D were found to have similar concentrations to those obtained in the third phase. On the other hand, sampling point C was found to have a higher concentration compared to the previous value. Thus, it seems that some error affected the first measurement of sample C. The histogram for the 100 samples obtained in this case is again positively skewed, similar to those of the previous phases (Figure 5d). Figure 6d illustrates the spatial distribution of the Gi* *z*-scores. The samples are evenly distributed overall, except for those in the southwest region of the study area. This region was not regarded as significant (being a cold spot region), based on the hot spot analysis and group classification of each phase. The statistical summary of the transformed PXRF data for the 100 sampling points yielded minimum, maximum, mean, and std. dev. values of 40, 5477, 771.8, and 1027.4 mg/kg for the copper concentration, respectively. The Gi* *z*-scores yielded minimum, maximum, mean, and std. dev. values of −2.05, 3.76, 0.50, and 1.73 via hot spot analysis, respectively (Table 2). Figure 7d shows the scatter plot of the 100 samples classified into the four groups. Despite the re-investigation of sampling point B, sample B was again classified as class 4, as for the third-phase result. On the other hand, the other three class-4 samples identified in the third phase, which were located near the *y*-axis (Figure 7c), were re-classified as class 1 or 3 based on the slightly altered Gi* *z*-scores and normalized concentrations by additional surveys. Sample C was reclassified from class 2 to class 1 according to the re-investigation result. Further, although similar concentration values were obtained for sample D in the first and second investigations (phases 3 and 4; Table 3), this sample was also reclassified from class 2 to 1. This change was made because the normalized concentration value of sample D increased slightly, as the concentration mean decreased from 895.6 to 771.8 mg/kg. The spatial distribution of the group classification is shown in Figure 8d. If additional surveys are planned, an investigation of the area west of sample B is one suggestion for the next phase.

As demonstrated by the above results, the group classification method suggested in this study can be utilized to plan a soil survey procedure and to identify samples that require a complementary investigation. In phases 1 and 3, more samples (30) were investigated to expand the survey area. In phases 2 and 4, fewer samples (20) were investigated, as the aim was to re-investigate the pre-surveyed area. A simple and intuitive method using four quadrants can be applied to classify the samples, as detailed above. However, the classes of the samples located near the scatter-plot borders can be easily changed by slight variations. This may be a common phenomenon for samples near the scatter-plot origin in particular. Thus, other methods that consider the gradient or the distance from the origin in the scatter plot may be employed in the classification stage in future applications. Nevertheless, the method presented herein can provide very useful and intuitive information on samples. In this study, a small number of class-4 samples were obtained, and some of these samples were even reclassified as other classes following an additional survey. Therefore, samples of this type requiring a complementary investigation can be found easily. In addition, class-2 samples can be further categorized into one of two types: those at the spatial boundary of a class-1 cluster (an explicit hot spot) and those underestimated because of certain errors, as exemplified by sample C above. Because the former is a much more common case and as these two sample types are not segregated in the scatter plot, the spatial distribution map of the group classification should be examined to locate samples misclassified because of some error.

## 4. Conclusions

In this study, Getis-Ord Gi* hot spot analysis and a proposed group classification method were employed to map copper-contamination hot spots in a given study area, and to support the development of soil sampling plans. Element analysis data obtained via PXRF instruments and validated by ICP-AES were used. With the development of the PXRF instrument, which is relatively cost and time effective, a considerably greater number of sampling points can be investigated instantly. Therefore, it is possible to plan the next investigation step rapidly based on an analysis of the acquired data. However, the relatively low accuracy of PXRF data or investigation errors can be a source of error in the data. Herein, hot spot analysis and the proposed group classification method were applied to each soil sampling phase, not only to identify the hot spot areas, but also to identify samples suspected to have an error. 

Comprehensive site investigation work, including movement, soil sample analysis, data transmission, hot spot analysis, group classification, and subsequent soil sampling planning, was conducted for 2 d, from 9 am. to 5:30 pm. Overall, 170 min were required to analyze the copper concentrations at the 30 sampling points of phase 1, and approximately 80 min were required to analyze the data and plan the next phase, including the breaks. A roughly 120-min period was spent analyzing the copper concentrations at the 20 sampling points of phase 2. Again, approximately 80 min were required to analyze the data and plan the next phase. Phases 1 and 2 were conducted on the first day and phases 3 and 4 were conducted on the second day. The durations of the second-day tasks were similar to those of the first day. Note that the soil investigation and mapping conducted solely via ICP-AES, which is the conventional method used in the geochemistry field, may require a period of at least 7 d [29]. Therefore, the developed method using PXRF can not only help plan the soil sampling, but also substantially reduce the implementation time.

Although numerous previous studies utilizing geostatistical interpolation to predict PTE spatial variation in soil have been reported, the methods used in those studies cannot provide information on the statistical significance of each sample and the potential measurement errors. Geostatistical interpolation methods are very useful for assessing the spatial distribution of contamination by predicting the unknown values for any location. However, difficulties arise when these methods are used to find a defective sample before contamination mapping. Hot spot analysis is not used to predict unknown values; however, this approach can be used to acquire better-quality samples before contamination mapping by assessing the statistical significance of each sample in the field. The results of this study confirm that hot spot analysis can provide information on the statistical significance of each sample. This information is important for sample assessments and for the planning of the next soil sampling process. The application of hot spot analysis and the proposed classification approach can constitute a valuable filter for the identification of critical areas in soil sampling. If the method proposed in this study is used in conjunction with geostatistical interpolation, it may contribute to a remarkable improvement in soil contamination mapping from the perspective of reliability.

The spatial variation of PTEs in soil can be heavily affected by topography and surface erosion due to runoff, unless appropriate isolation or treatment measures are implemented [36,37,38,39]. Therefore, in future research, a consideration of the surface flow when specifying the distance from each feature to neighboring features may be worthwhile. If software is developed to allow the application of the method presented in this study and embedded in the PXRF instrument, the latter can become a very useful tool for the development of sampling plans in the field. In addition, it is expected that hot spot analysis can be applied to 3D geochemical data for mineral exploration or the detection of underground pollution.

## Figures and Tables

**Table 1:** Copper concentration results determined using ICP-AES and PXRF for 12 sampling points.

No.	Cu (mg/kg) (ICP-AES)	Cu (mg/kg) (PXRF)	Cu (mg/kg) (Transformed PXRF)	Remark
1	17	29	45	12 sampling points at which both ICP-AES and PXRF analyses were performed
2	29	40	52	
3	33	58	64	
4	48	78	77	
5	57	94	88	
6	344	499	355	
7	636	1044	715	
8	1080	1470	997	
9	1338	1731	1169	
10	1535	2316	1555	
11	4041	5853	3891	
12	4437	6930	4602	

**Table 2:** Descriptive statistics of the transformed copper concentration and Gi* *z*-score results according to the four-phase procedure.

Item	Phase	The Number of Samples	Minimum	Maximum	Mean	Std. Dev.
Transformed copper concentrations	1	30	45	4602	961.6	1121.5
	2	50	45	4602	902.8	958.0
	3	80	40	5477	895.6	1102.2
	4	100	40	5477	771.8	1027.4
Gi* *z*-score	1	30	−1.36	1.78	0.18	0.86
	2	50	−2.16	3.38	0.29	1.42
	3	80	−2.26	3.56	0.41	1.61
	4	100	−2.05	3.76	0.50	1.73

**Table 3:** Re-investigation results for sampling points suspected to be influenced by error.

Sampling Point	Initial Analysis Phase (Phase)	Initial Cu Value (mg/kg)	Initial Classification (Class)	Second Analysis Phase (Phase)	Second Cu Value (mg/kg)	Second Classification (Class)
A	1	1505	4	2	112	3
B	3	1320	4	4	1333	4
C	3	353	2	4	1555	1
D	3	865	2	4	838	1

## Abbreviations

The following abbreviations are used in this manuscript:

PXRF	Portable X-ray fluorescence
ICP–AES	Inductively coupled plasma atomic emission spectrometry
PTEs	Potentially toxic trace elements
GIS	Geographic information systems
HH	A high concentration value with a high *z*-score
LH	A low concentration value with a high *z*-score
LL	A low concentration value with a low *z*-score
HL	A high concentration value with a low *z*-score
